# Age-Associated Splicing Events in the Integrative Longevity Omics (ILO) Cohort

**DOI:** 10.1093/geroni/igaf122.535

**Published:** 2025-12-31

**Authors:** Payton Bock, Stefano Monti, Paola Sebastiani, Thomas Perls, Stacy Andersen, Eric Reed, Albert Tai

**Affiliations:** Boston University, Boston, Massachusetts, United States; Boston University, Boston, Massachusetts, United States; Tufts Medical Center, Boston, Massachusetts, United States; Boston University, Boston, Massachusetts, United States; Boston University, Boston, Massachusetts, United States; Albert Einstein College of Medicine, New York City, New York, United States; Tufts University, Boston, Massachusetts, United States

## Abstract

Alternative splicing regulates transcript diversity and is increasingly recognized as a key mechanism in aging and age-related disease, yet little is known about how splicing contributes to human exceptional longevity. We analyzed RNA-seq data from the Integrative Longevity Omics (ILO) cohort, which includes centenarians (the oldest-old, age ≥100) and older adult controls (ages 60–89), to identify age-associated splicing events. Splicing was quantified with rMATS to estimate percent-spliced-in (PSI) values across five event categories, and quasi-binomial regression was used to model splicing changes with age. This analysis identified 731 significant age-associated events (false discovery rate < 0.05), the majority of which were mutually exclusive exons and skipped exons. A strong example is a skipped exon in Nuclear Transcription Factor Y subunit C (NFYC), a regulator of senescence-associated pathways. In controls, NFYC PSI trended downward with age, whereas in centenarians, PSI increased significantly with age, suggesting a reversal of midlife splicing trajectories. Residual analysis comparing observed PSI to predictions from a spline model of normal aging further suggests that centenarians deviate from normal aging patterns. This work describes age-associated splicing events in a population enriched for centenarians and provides preliminary evidence for a candidate splicing signature of exceptional longevity. Ongoing analyses will evaluate functional pathways and clinical relevance in independent cohorts.

